# Morphological and Genomic Differences in the Italian Populations of *Onopordum tauricum* Willd.—A New Source of Vegetable Rennet

**DOI:** 10.3390/plants13050654

**Published:** 2024-02-27

**Authors:** Simona Casavecchia, Francesco Giannelli, Massimo Giovannotti, Emiliano Trucchi, Federica Carducci, Giacomo Quattrini, Lara Lucchetti, Marco Barucca, Adriana Canapa, Maria Assunta Biscotti, Lucia Aquilanti, Simone Pesaresi

**Affiliations:** 1 Department of Agriculture, Food and Environmental Sciences, Marche Polytechnic University, Via Brecce Bianche, 60131 Ancona, Italy; g.quattrini@pm.univpm.it (G.Q.); l.lucchetti@pm.univpm.it (L.L.); l.aquilanti@univpm.it (L.A.); s.pesaresi@univpm.it (S.P.); 2 Department of Life and Environmental Sciences, Marche Polytechnic University, Via Brecce Bianche, 60131 Ancona, Italy; m.giovannotti@univpm.it (M.G.); e.trucchi@univpm.it (E.T.); f.carducci@univpm.it (F.C.); m.barucca@univpm.it (M.B.); a.canapa@univpm.it (A.C.); m.a.biscotti@univpm.it (M.A.B.)

**Keywords:** vegetable rennet, ddRAD sequencing, morphometric data analysis, Taurian thistle, populations genetic, genotyping-by-sequencing

## Abstract

*Onopordum tauricum* Willd., a species distributed in Eastern Europe, has been the subject of various research endeavors aimed at assessing its suitability for extracting vegetable rennet for use in the production of local cheeses as a substitute for animal-derived rennet. In Italy, the species has an extremely fragmented and localized distribution in six locations scattered across the central-northern Apennines and some areas of southern Italy. In this study, both the morphology and genetic diversity of the six known Italian populations were investigated to detect putative ecotypes. To this end, 33 morphological traits were considered for morphometric measurements, while genetic analysis was conducted on the entire genome using the ddRAD-Seq method. Both analyses revealed significant differences among the Apennine populations (SOL, COL, and VIS) and those from southern Italy (ROT, PES, and LEC). Specifically, the southern Italian populations appear to deviate significantly in some characteristics from the typical form of the species. Therefore, its attribution to *O. tauricum* is currently uncertain, and further genetic and morphological analyses are underway to ascertain its systematic placement within the genus *Onopordum*.

## 1. Introduction

For ethical reasons related to the respect for animals, an increasing number of plant-based ingredients are being used in food manufacturing, which traditionally relied on animal derivatives (such as soy milk or milk from other plant species, soy burgers, cheeses made with vegetable rennet, etc.). The demand for vegetable rennet in the production of cheeses is increasing because of: (i) decreasing availability of calf rennet [1]; (ii) religious restrictions (e.g., Judaism, Islam, Buddhism); (iii) diet preferences (vegetarianism), and (iv) health-related reasons, such as the high incidence of bovine spongiform encephalopathy [2]. Several species of the Asteraceae family, belonging to different genera such as *Cynara* (*C. cardunculus*, *C. scolymus*, *C. humilis*), *Sylibum* (*S*. *marianum*), *Centaurea* (*C*. *calcitrapa*), *Cirsium* (*C*. *vulgare*), and *Onopordum* (*O. acanthium*, *O. nervosum* ssp. *platylepis*, *O. turcicum*), are traditionally used in cheesemaking and have been investigated to isolate clotting enzymes [3]. Recently, it has been demonstrated that *O. tauricum* has the potential to produce vegetable rennet for use in the production of local cheeses [4,5,6]. In connection with this, the Taurian thistle has also been evaluated as a candidate species for cultivation [7].

*Onopordum* is an angiosperm genus belonging to the family Asteraceae. The native range of this genus extends from Kazakhstan and Kirgizstan—in central Asia—to western Europe (Iberian Peninsula) and northern Africa (from the Canary Islands and Morocco to Egypt). It also occurs in the Arabian Peninsula and in northern Europe (Norway and Sweden). Some species of *Onopordum* have been accidently introduced to Great Britain and Ireland, the United States, Canada, Australia, and Tasmania [8], where they are regarded as noxious species kept under strict control because of their weed potential [9,10]. All species belonging to this genus are biennial: at the end of the summer, with the first rains, seeds give rise to seedlings that develop into large rosettes, which stay in this form for the whole winter. In the spring season, with increasing daylight duration, these rosettes develop into an adult plant that can bear, in terminal position, several flower heads. As they tolerate periods of aridity and cool winters, these plants inhabit regions characterized by a Mediterranean climate, where they occur in pastures, rocky areas, abandoned fields, and ruderal areas. Soils with abundant nitrates favor the presence and growth of *Onopordum*.

The phylogeny of the genus *Onopordum* is largely controversial, and the meaning of the informal group *Onopordum* within the subtribe Carduinae (Tribe Cardueae) has been long investigated using both nuclear ribosomal DNA and chloroplast DNA markers [11,12,13,14,15]. Recently, Herrando-Moraira et al. [16] resolved the phylogeny of this genus and established the subtribe Onopordinae, which includes the genera, *Onopordum*, *Alfredia*, *Syreitschikovia* and *Olgaea*, by a Hyb-Seq approach. It is believed that all the species included in the genus have a recent origin as they probably diverged in rapid and explosive speciation during the Pliocene–Pleistocene, subsequent to the appearance of the Mediterranean climate [15,17,18,19].The genus currently includes 60 accepted species, five of which occur in Italy: *O. acanthium* L., *O. illyricum* L., *O. tauricum* Willd., *O. horridum* Viv., and *O. macracanthum* Schousb.

The Taurian thistle is up to 2 m tall and is more or less viscid due to the occurrence of glandular hairs covering its leaves, stems, and flower heads. As all species of the same genus, the stem is winged all along, with wings up to 15 mm wide and spines up to 5 mm long. The Italian range of this species is fragmented and characterized by small populations with localized distributions. It occurs in small, fragmented populations in the Marche Region, the Tuscan–Emilian Apennines, the Gargano Peninsula, Southern Molise, and Salento Peninsula (Apulia Region), as recorded in Acta Plantarum [20]. Therefore, it can be hypothesized that the Italian population of *O. tauricum* is a metapopulation compared to the main nucleus of the species, being distributed at the western margin of the species’ native range. Indeed, it is likely that its presence in France may result from accidental introduction [21]. This species occurs in synanthropic habitats, in the presence of domestic animals, especially sheep. It is commonly found near stables, in pastures, and in cultivated and abandoned fields. It is a nitrophilous species and therefore prefers soils rich in nitrates and organic matter. Different varieties of *O. tauricum* were described in the past for the Italian range. Some of these varieties are currently regarded as synonyms of the nominal species or attributed to other species. In addition to the *typicum* variety, the following varieties are currently reported: variety *elatum* Sm. for Sicily (based on a single report in the area of Milazzo), subsequently recognized as a synonym of *O. argolicum* Boiss., which was in turn recognized as a synonym of *O. tauricum* variety *apulum* Fiori, described for some localities of Apulia and other localities in southern Italy, subsequently included in *O. horridum* variety *horridum* Viv. [22] recognized for southern Italy, and subsequently included, by synonymy, in *O. horridum* Viv. [23,24].

In order to successfully cultivate wild species, a deep knowledge of all aspects of plant biology and ecology is necessary. Indeed, our knowledge of wild plants in terms of their biology is rather poor, and deepening the knowledge of their genetics and genomic traits can be regarded as the first step in evaluating possible strategies for their domestication, also with reference to the geographic origin of the individuals to be cultivated and the area where their cultivation will be carried out. Genetic improvements in wild plants for cultivation purposes could be achieved through a clear understanding of the plant’s ecology and the extent of variability within wild populations, including genotypes that may hold great potential for adaptation to different ecological conditions. Therefore, the identification of “ecotypes” could be important in choosing which germplasm should be used in a certain geographic area. Moreover, Italian populations occur at the western limit of the native distribution range of the species so that different climatic and environmental condition can exert strong pressure toward the adaptation and appearance of specific characteristics or even differentiation at the taxonomic level.

In the present research, the diversity of six Italian populations of this species was investigated using a ddRAD-seq approach and the analysis of 33 morphological traits, with the aim of assessing the occurrence of different ecotypes.

## 2. Results

### 2.1. Genetic Structure Analysis 

**Figure 1:**
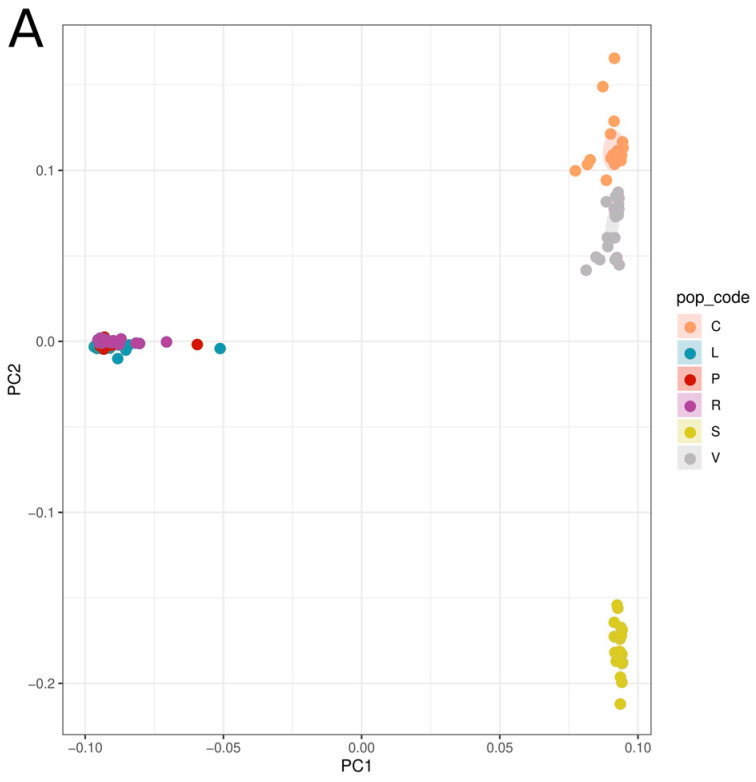

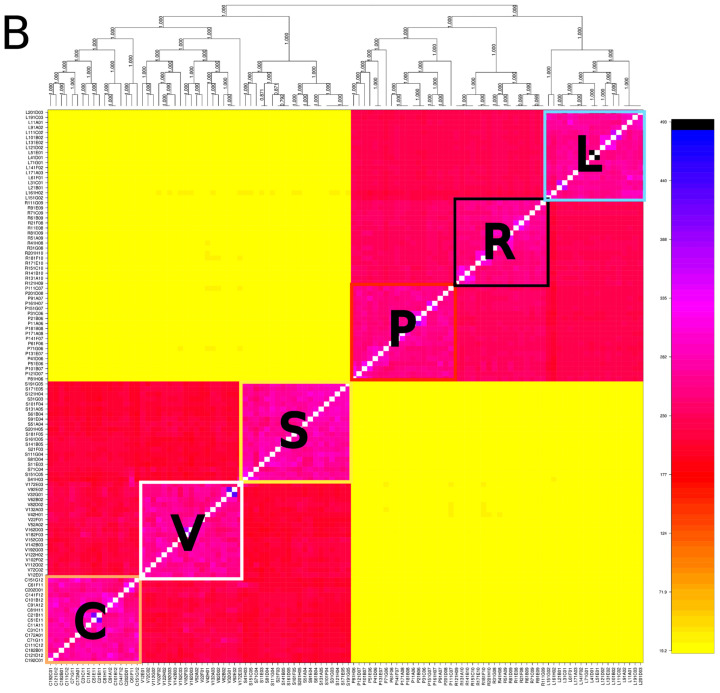
PCA plot (**A**) and co-ancestry matrix (**B**) analyses, both showing clear separation between the north-central and southern clusters. COL (C), VIS (V), SOL (S), PES (P) ROT (R), LEC (L). PCA axis 1 (PC1) accounts for 63.33% of the variation, while PCA axis 2 (PC2) accounts for 5.73%.

The final datasets, with all populations included, comprised 133,343 SNPs in 18,291 loci, whereas the SOL-COL-VIS cluster dataset included 129,195 SNPs in 41,950 loci, and the LEC-PES-ROT cluster dataset included 122,159 SNPs in 42,689 loci, respectively. Both PCA and fineRADstructure analyses clearly revealed the presence of two main clusters, with no evidence of gene flow between the two, perfectly representing the geographic distribution of the individuals (Figure 1). One cluster was located in northern-central Italy, including individuals from SOL, COL, and VIS localities, and another cluster was located in southern Italy, including individuals from LEC, PES, and ROT localities. Interestingly, PC2 (Figure 1A) separated samples from central Italy (C and V) from those of northern Italy (S).

Subpopulations within each cluster show a high level of gene flow, but they still appear as distinct genetic entities (Supplementary Figure S1). This separation between the two clusters is also supported by F_ST_ values (Table 1), varying from 0.43, between the LEC population and the VIS population, and 0.54, between the SOL population and the PES population. F_ST_ supports a more consistent gene flow within the southern cluster than within the northern-central one; the highest F_ST_ value is 0.07 (between PES and LEC in the southern cluster), while F_ST_ ranges from 0.08 (between COL and VIS) to 0.13 (between SOL and COL) in the northern-central cluster. Regarding the genetic diversity statistics (Table 2), we found that the population showing the highest genetic diversity is VIS (π = 2.5 × 10^−3^), whereas the lowest genetic diversity is observed in PES (π = 1.5 × 10^−3^).

### 2.2. Phylogenetic Tree

In the phylogenetic tree (Supplementary Figure S2), sequences of *O. tauricum* are subdivided into two subgroups: one including sequences of SOL, COL (with the exception of C5 and C15), and VIS; and the other comprising sequences of LEC, ROT, and PES. However, this separation was not supported by a significant posterior probability value. Notably, a clear distinction between sequences of *O. tauricum* and those belonging to other species was not highlighted. The sequences of *O. horridum* and *O. acanthium* grouped with those of *O. tauricum* sampled in Sologno, Colfiorito, and Visso, while the sequences of *O. seravschanicum*, *O. illyricum*, *O. nervosum*, *O. carduchorum*, *O. anatolicum*, and *O. caricum* seem to be correlated with those of *O. tauricum* sampled in Lecce, Peschici, and Rotello sites.

### 2.3. Morphometric Characterization

**Figure 2:**
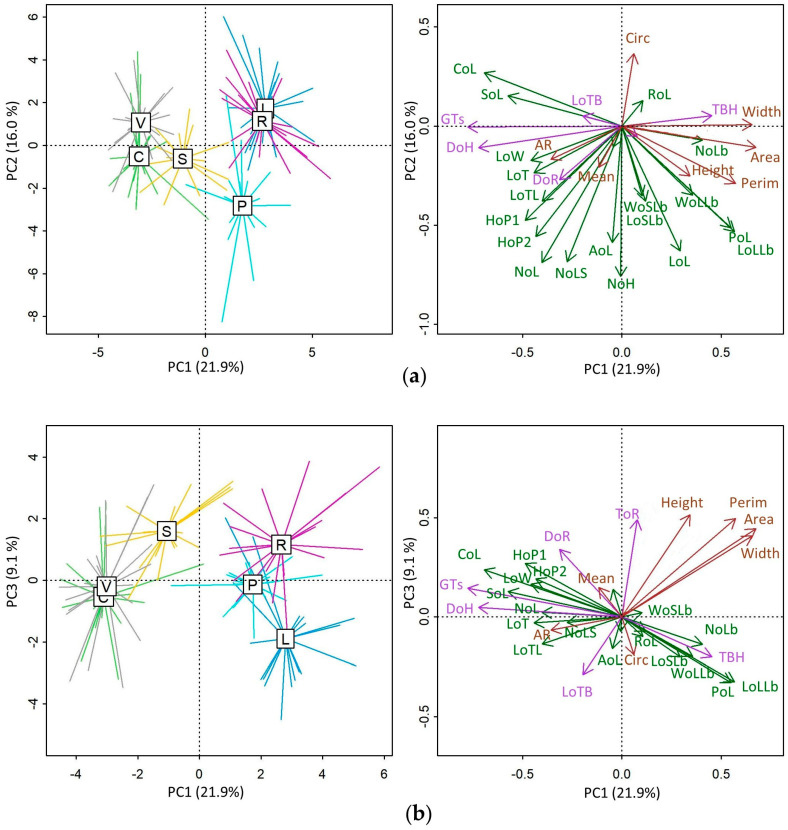
Principal component biplot relating morphometrics traits (arrows) to the six Italian populations of *Onopordum tauricum*. PCA axis 1 accounts for 21.9% of the multivariate variation, while PCA axis 2 and PCA axis 3 account for 16.0% and 9.1%, respectively. (**a**) PCA ordination space defined by PC1 and PC2 axis. (**b**) PCA ordination space defined by PC1 and PC3 axis. Population labels: COL (C), VIS (V), SOL (S), ROT (R), PES (P), LEC (L); morphometric trait labels are those listed in Material and Methods and grouped in distinct colors: in green, traits related to the whole plant and leaves; in brown, traits related to the fruits; in purple, traits related to the flower heads.

The results of measurement, counting, and calculated ratios are shown in Supplementary Table S1. Standardized PCA allows for a visual representation that facilitates Italian populations’ morphometric comparison. Similar populations are close together, and dissimilar populations are further apart. Different populations are represented in spider plots. The morphometric traits are indicated by arrows (Figure 2). The first three principal component axes account for 21.9% PC1, 16.0% PC2, and 9.1% PC3, respectively. The six Italian populations of *O. tauricum* are clearly separated into two groups along the PC1 axis: the first group represents northern and central Apennine populations (SOL, VIS, and COL), while the second group includes populations from southern Italy (ROT, PES, and LEC).

The morphometric traits characterizing the two groups along the first axis are: (i) occurrence of glandular trichomes (GTs) on the bracts of the flower heads; (ii) diameter of the flower heads (DoH); (iii) diameter of the receptacle (DoR); (iv) length of the spine of the bracts of the flower heads (LoTB); (v) length of the stem wing (including the spine, LoW); (vi) length of the wing spine (LoT); (vii) length of the leaf spine (LOTL); (viii) circularity of leaf (CoL); (ix) solidity of leaf (SoL); (x) height of the main plant stem (HoP1). The values of these traits are higher in the Apennine populations. On the contrary, the traits whose values are higher in the southern populations are: (i) length of the middle leaves (LoL); (ii) length of the longest lobe of the leaf (LoLLb); (iii) width of the longest lobe (WoLLb); (iv) number of leaf lobes (NoLb); (v) occurrence of non glandular trichomes in the flower heads bracts (TBH); (vi) leaf perimeter (PoL); (vii), achenes characters (height, BX, BY, perimeter, area, and width).

The PERMANOVA (F = 36.46, R^2^ = 0.61, *p* < 0.001, 9999 permutations) revealed significant differences in the multivariate space defined by the three PCA components (see Figure 2). Furthermore, the post hoc analysis of the PERMANOVA indicated that all populations were significantly different from each other, except for the COL-VIS pair (Supplementary Table S2).

**Figure 3:**
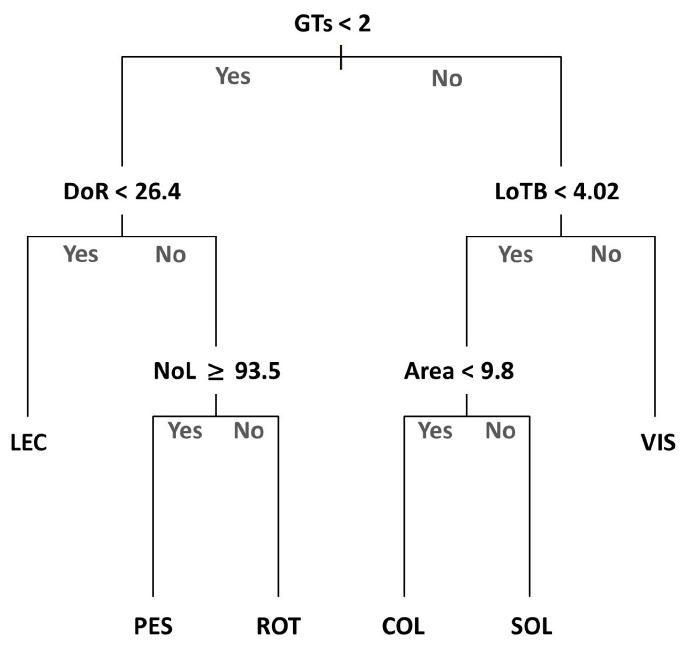
Classification tree showing the relationship between the six populations of *Onopordum tauricum* and key predictive morphometric characteristics that include GTs (Glandular trichomes), DoR (diameter of the receptacle), NoL (number of leaf lobes), LoTB (length of the spines of the flower heads), and Area (area of the achenes).

The classification tree model had an overall accuracy of 78.41% (±11.73), with the best cp value being 0.025 (see Supplementary Table S3 for the confusion matrix). The tree pinpointed the abundance of glandular trichomes in the flower head bracts (GTs) as the key feature distinguishing the Apennine and southern populations (Figure 3). Within the Apennine group (COL, VIS, SOL), the length of the spines of flower head bracts (LoTB > 4.02 mm) discriminates VIS from COL and SOL, while the area of the seeds differentiate COL from SOL (whose seeds are >9.831 mm^2^). For the second group, the diameter of the receptacles of flower heads (DoR < 26.44 mm) discriminates LEC from PES and ROT, with the latter populations differing from each other in the number of leaves (NoL).

## 3. Discussion

As mentioned in the introduction paragraph, the Taurian thistle is one of the candidate species for the production of vegetable rennet to be used in the production of local cheeses [4,5,6,7], and its suitability was tested during the activities of the European Project PRIMA “Valorisation of thistle-curdled CHEESES in MEDiterranean marginal areas” (acronym “VEGGIE-MED-CHEESES”), aimed at finding suitable plants.

Both genomic (Figure 1) and morphological (Figure 2) analyses suggest that the putative Taurian thistle populations occurring in Italy belong to two different taxonomic units. Use in cheesemaking has been tested [4,5,6] only for the population from Colfiorito and Visso (COL and -VIS genomic cluster), and it is therefore deemed necessary to also assess specimens from the other genomic cluster.

**Figure 4:**
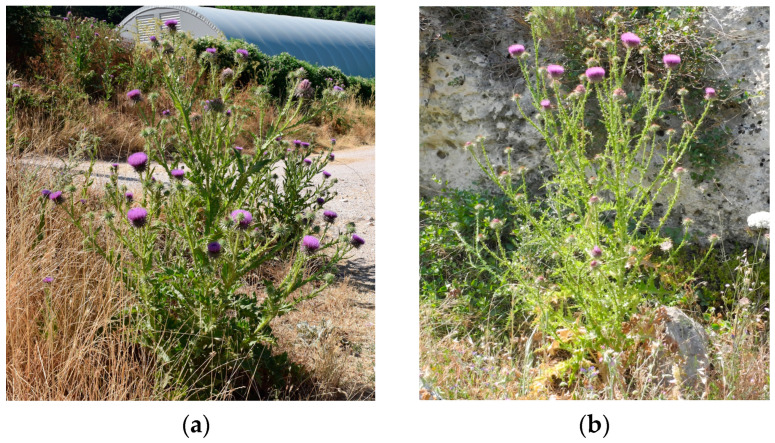
*Onopordum tauricum* Willd. from Cupi di Visso (**a**) and Lecce (**b**).

The general appearance of the plant (Figure 4), especially with regards to its indument, leaf morphology, and some characteristics of the flower head, differs quite evidently between the central-northern populations (SOL, VIS, and COL) and the southern ones (ROT, PES, and LEC). These differences were also confirmed by morphometric analysis. In particular, the most evident and stable characteristic that differentiates the two groups of population is the abundance of glandular trichomes (GTs) over the entire body of the plant. Indeed, individuals from the central and northern Apennine populations are overall sticky, while those from the southern populations are much less so.

It is not known whether environmental conditions can affect glandularity. The ability to develop glandular trichomes (GTs) is in fact considered a genetically controlled trait, even if the regulatory mechanisms are poorly understood [25,26,27]. As is known, GTs are epidermal structures specializing in the synthesis of metabolites, enabling plants to adapt to both abiotic and biotic environmental stresses. Two different types of GTs have been described [28]: peltate trichome and capitate trichome. In all the Italian populations investigated, GTs belong to the second type, consisting of a multicellular stalk with a smaller unicellular head. 

In addition to the GTs, non-glandular trichomes, which are long, white, and multicellular, occur on the leaves and the lower bracts of the flower heads. They are very abundant on the abaxial side of the leaves, where they form a whitish felt, and in the basal bracts of the flower heads of the populations of southern Italy, while in the Apennine populations, they are rather rare and concentrated in the central vein of the leaves. In the literature, the presence of dense white trichomes on the body of the plant is related not only to the plant’s defense against excessive solar radiation and high temperatures [29,30] but also to defense against herbivores. The dense whitish hairiness, in fact, represents a possible mimicry of spider silk webs or fungal hyphae, which could act as a deterrent against herbivores [31]. The presence of whitish and cobwebby hairs in the covering of the flower head that we found in individuals of the populations of southern Italy could therefore represent a defense against herbivores, which compensates for the absence or scarcity of glandular trichomes, which are, on the contrary, abundantly present in populations in Central and Northern Italy.

Further morphological characteristics that clearly distinguish the northern populations from the southern ones are the diameter of the flower heads and receptacles, which are significantly greater in the former. This dimensional characteristic, although potentially dependent on environmental conditions and, above all, on the richness of nutrients present in the substrate, is very stable within populations and between geographical areas. Considering that the habitats in which the plants live are similar for all sampled populations, this is considered a discriminating characteristic between populations in the central north and those in the south.

The same considerations can be made with reference to the height of the main stem (height), which appears to be greater in the populations of southern Italy.

The statistical comparison highlighted a significant difference in the length of the spines on the bracts of the flower heads, leaves, and wings of the stem between the Apennine populations and the southern ones. In the scientific literature, the presence of spines, thorns, or prickles on plant bodies is reported as a physical defense [32,33,34,35,36,37,38] and microbiological defense [39,40,41,42] that plants implement against herbivores. Therefore, plants that live in environments frequented by herbivores are particularly equipped with it [43,44]. Many thorny plants belonging to the Asteraceae family, including *Onopordum* and other thistle species, live in grazed areas and are particularly abundant near stables and in areas where animals stay for a long time, as they benefit from the nitrates and organic substance provided by animal excrement. Thanks to their “arsenals” of spines, they are avoided by most herbivorous animals, except donkeys, which, according to tradition, are greedy for them. It can therefore be deduced that the abundance of spines in many organs (stem, leaves, and flowerheads) represents a characteristic that has emerged as an adaptation to environmental conditions [45]. The defense function is not only due to the length of the spines but above all to their robustness. Therefore, differences in the length of the spines we found are probably not due to selective pressure but could be linked to distinct genetic regulators [46]. Asteraceae, with spines occurring in the Flora of Israel, were the subject of a study that led to the identification of five different protective spiny modules represented by: (i) spiny rosette leaves, (ii) spiny cauline leaves of the stems and branches, (iii) spiny wings of stems and branches, (iv) spiny inflorescence heads, and (v) thorny branch tips [45]. *Onopordum tauricum* and, more generally, all the species belonging to the genus *Onopordum* present four of the five spiny modules listed above, namely spiny rosette leaves, spiny cauline leaves of the stems and branches along the entire perimeter of the leaf, spiny wings of stems and branches, and spiny bracts of inflorescence heads. Thorniness is therefore a constant and very abundant characteristic throughout the body of the plant. It is significant that our measurements of the spines in various Italian populations highlighted the greater length of the spines on modules 2-3-4 in all the populations of central and northern Italy compared to those in southern Italy.

Other important differences between the two groups of populations concern leaf morphology. In particular, the median cauline leaves of the populations in central and northern Italy are shorter (LoL) and less incised (>degree of circularity and solidity). Circularity is the ratio of the leaf area to the perimeter of the outline, and therefore, it is sensitive to the number of lobes and serration of the leaf. Indeed, the median cauline leaves of the ROT, PES, and LEC populations (southern Italy) present a greater length (LoLLb) and width (WoLLb) of the lobes and, being longer and more etched, they have a higher number of lobes (NoLb) and a longer perimeter (PoL).

Leaf morphology is a widely studied topic, as the shape of the leaf and its size considerably influence the photosynthetic yield and commercial value of many cultivated plants (e.g., [47,48,49,50,51,52,53,54,55,56,57]). Therefore, over the years, numerous studies have been conducted, aimed at identifying and mapping the genes (QTLs) responsible for the size and shape of leaves and carefully analyzed on a morphometric basis [58,59,60,61] through transcriptional analysis methods. However, the genetic architecture and molecular regulatory mechanisms upon which leaf morphology depend have not been completely clarified yet. Several studies have clarified how environmental conditions (light, temperature, water availability, salt, UV radiation, heavy metals, etc.) can modify the factors of transcription and regulation of gene expression [62,63,64,65,66,67].

Significant differences between the two groups of populations were finally found in the size (height, width) and shape (BX, BY, perimeter, area) of the fruits. Indeed, micro- and macromorphological characteristics of Asteraceae achenes have been used for the identification and systematics of numerous genera [68,69,70,71,72,73,74,75,76] and have been found to be very useful for taxonomic diagnosis in many critical genera, such as *Centaurea* [74,77,78,79,80,81,82,83,84,85,86,87]. The characteristics that were found to be more diagnostic are: shape, size, pericarp texture and color, surface structure, hilum position, and pappus hairs.

In regards to the correlations between morphology and genetics, numerous studies have been conducted on species of food interest (i.e., tomato, cucumber, chickpea, summer squash, etc.), which have made it possible to establish that fruit shape is quantitatively inherited and numerous QTLs genes have been isolated [88,89,90,91,92,93,94,95,96,97]. This suggests that the differences in achenes detected between groups of populations could have systematic significance, although further investigations are necessary.

The above considerations seem to suggest that the populations of southern Italy could be attributed to a different taxon. In this regard, a putative candidate could be *O. horridum* Viv., described for Corsica and Sardinia and later reported for other regions of southern and central Italy as well [23,98,99]. However, on the basis of the morphological characteristics reported in the literature and considering the protologue upon which the description of *O. horridum* is based [100], it is not possible to attribute ROT, PES, and LEC populations to this species. Phylogenetic analysis (Supplementary Figure S2) also highlights that the sequences belonging to individuals of southern Italy (LEC, ROT, and PES) are more genetically distant from *O. horridum* than those of individuals in northern Italy.

Moreover, it is not possible to establish if the northern and southern populations of *O. tauricum* were separated before or after their introduction to Italy. Overall, the mode and time of arrival of this species in Italy remains unclear. The fruits of *O. tauricum* probably arrived in the Italian Peninsula repeatedly with the transhumance of flocks.

Actually, systematic attribution at a specific level for the different *Onopordum* entities is very difficult due to both the high morphological variability within the species and a certain degree of ambiguity or lack of clarity in the available dichotomous keys. Indeed, over the years, some taxonomic and systematic reviews have been published discussing possible hybrids between different species [101,102,103,104,105,106,107,108]. On the other hand, new species of *Onopordum* are continuously described [109,110,111,112,113,114], testifying to the fact that the *Onopordum* genus is still in the process of establishing itself. It is probable that disturbance due to environmental pressures, including anthropic activities, can favor the speciation process in biennial species and those of polyploid origin, as suggested by Garcia-Jacas et al. [17].

Therefore, it is necessary to further investigate the systematics of the populations in southern Italy and, to this end, an in-depth comparative study is underway, aimed at verifying the opportunity to re-evaluate the variety *apulum* Fiori, already described and currently considered a synonym of *O. horridum*. At the moment, therefore, the populations of southern Italy are considered as “ecotypes” belonging to the “*O. tauricum* Group” while awaiting a more certain systematic placement.

## 4. Materials and Methods

### 4.1. Species Identification

The currently known Italian distribution range of *Onopordum tauricum* includes the regions Marche and Umbria (National Park of Sibillini Mounts) [22,23]. More recently, the species was found in Emilia–Romagna at the locality Sologno (Reggio Emilia), in Molise at the locality Rotello (Campobasso), and in the Salento peninsula close to Lecce, as recorded in Acta Plantarum https://www.actaplantarum.org/ (accessed on 15 January 2024). The identification of the sampled plants was carried out according to the dichotomous keys in Flora Europaea [115] and in the Italian Flora [23], and on the basis of morphological traits indicated as diagnostic, such as: the stem and leaves’ indumentum (the plants have a bright green color due to the lack of protective hairs on the stem and leaves) and the occurrence of glandular trichomes on the flower head bracts.

### 4.2. Plant Collection and DNA Isolation

**Figure 5:**
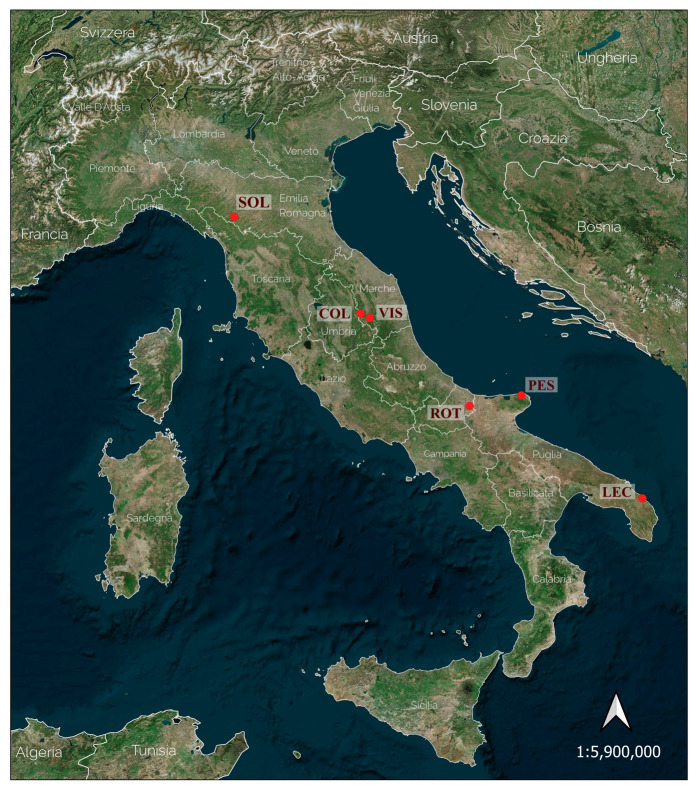
Localization of the sampled populations (SOL = Sologno (RE); VIS = Cupi di Visso (MC); COL = Colfiorito (PG); ROT = Rotello (CB); PES = Peschici (FG); LEC = Lecce).

From north to south, the sampled populations are: Sologno (henceforth, Population SOL); Visso (henceforth, Population VIS); Colfiorito (henceforth, Population COL); Rotello (henceforth, Population ROT); Peschici (henceforth, Population PES); Lecce (henceforth, Population LEC). Figure 5 shows the geographic distribution of the sampled populations.

The SOL population was collected at the end of July in the Emilia–Tuscany Apennines within the municipality of Reggio Emilia, in a hilly area of about 800 m a.s.l. It was found in a shrubby meadow recently abandoned by livestock grazing.

The VIS population was sampled on the 20th of July in the Marche–Umbria Apennine, within the National Park of Sibillini Mounts (Macerata Province). The population occurs in a shrubby abandoned grassland next to a sheep pen, along a hill side at about 1000 m a.s.l.

Population COL is located in the Umbria–Marche Apennine, in the karst plateau of Colfiorito. This locality is not far from Population VIS (approximately only 16 km apart) but occurs in a parallel valley, separated by a large mountain range.

The ROT population is from a hilly area about 25 km far from the sea in southern Molise, at about 350 m a.s.l. The sampled population of *O. tauricum* was composed of about 50 plants widespread at the border of cultivated fields.

The PES population is located on the north side of the Gargano peninsula, in a flat area about 4 km from the sea. It is a small population of about 100 individuals growing in an abandoned field near some olive groves.

Finally, the LEC population is from Salento Peninsula. It is a big population of more than 100 individuals thriving in a grassland located not far from the sea and used as a pasture for sheep. (Table 3).

All the plants used for the analyses were in the same phenological stage, i.e., full flowering.

A total of 120 individuals (20 for each population, selected randomly in the population at a distance of no less than 5 m from each other) were sampled by collecting the two youngest leaves. The leaves were immediately dried by the immersion in a box filled with silica gel. In order to avoid cross-contamination among individuals, the leaves from each plant were placed in separate clean teabags [116]. Once the tissue was completely dry, we extracted DNA from 50 mg of dried leaf tissue using the DNeasy Plant Pro Kit by Qiagen, following the manufacturer’s instructions with some slight modifications.

### 4.3. RADseq Library Preparation and Sequencing

ddRAD libraries were produced using an IGATech custom protocol, with minor modifications with respect to Peterson’s double digest restriction-site associated DNA preparation [117]. The enzyme combination was selected by in silico analysis of related species of Cardueae. Genomic DNA were fluorometrically quantified, normalized to a uniform concentration, and 300 ng were double digested with 2.4 U of both NspI and MboI endonucleases (New England BioLabs, Ipswich, MA, USA) in a 30 µL reaction supplemented with CutSmart Buffer and incubated at 37 °C for 90′, then at 65 °C for 20′. Fragmented DNA was subsequently ligated with 200 U of T4 DNA ligase (New England BioLabs) to 2.5 pmol of overhang barcoded adapter for rare cut sites and to 5 pmol of overhang barcoded adapter for frequent cut sites in a 50 µL reaction incubated at 23 °C for 60′ and at 20 °C for 60′, followed by 20′ at 65 °C. Samples were pooled in multiplexing batches and purified with 1.5 volumes of AMPureXP beads (Agencourt). For each pool, the targeted fragment distribution was collected on a BluePippin instrument (Sage Science Inc., Beverly, MA, USA), setting the range of 350 bp–500 bp. Gel-eluted fraction were amplified with indexed primers using Phusion High-Fidelity PCR Master Mix (New England BioLabs) in a final volume of 50µL and subjected to the following thermal protocol [95 °C, 3′] − [95 °C, 30″ − 60 °C, 30″ − 72 °C, 45″] × 10 cycles − [72 °C, 2′]. Products were purified with 1 volume of AMPureXP beads. The resulting libraries were checked with both Qubit 2.0 Fluorometer (Invitrogen, Carlsbad, CA, USA) and Bioanalyzer DNA assay (Agilent technologies, Santa Clara, CA, USA). Libraries were sequenced with 150 cycles in paired-end mode on a NovaSeq 6000 instrument following the manufacturer’s instructions (Illumina, San Diego, CA, USA).

### 4.4. Identification of RAD Loci and SNP Calling

ddRAD raw reads were filtered, assembled into genomic loci, and SNPs were called using Stacks v.2.53 [118], following the standard pipeline for de novo assembly (as no reference genome was available). Assembly of short reads was performed using ustacks by setting -m 3 (stack default) and -M 5 parameters. For the compilation of the catalogue of genomic loci, the cstacks script was run with the -n 5 parameter (following Stacks recommendation of setting this parameter as the M parameter in the ustacks command). After genomic loci assembly and SNP calling, the script population in the Stack pipeline was used to extract the SNPs and perform population genetic analyses. Firstly, analyses were run using all 120 individuals divided into the six sampling localities, retaining only SNPs present in all localities (-p 6) and with no more than 25% missing individuals per locality (-r 0.75). We then ran the analyses again, separating the two main clusters LEC-PES-ROT and SOL-COL-VIS due to the high genetic distance revealed by the first analysis. In this case, loci were filtered using parameters -p 3 and -r 0.75, retaining loci present in all populations of each cluster and with no more than 25% missing individuals per population. The resulting datasets were exported as VCF and radpainter formats for downstream analyses. F_ST_ and basic genetic diversity statistics, like nucleotide diversity (π), expected (He), and observed heterozygosity (Ho), were also calculated using the population script in Stacks. To explore population structure, we implemented a Principal Component Analysis (PCA) using the SNPrelate [119] package in R and fineRADstructure v0.3.2 [120]. In the latter, we applied a stringent filter to the dataset, retaining only individuals with less than 10% missing data, which resulted in the exclusion of 10 individuals out of 120.

### 4.5. Phylogenetic Analysis

Phylogenetic analysis was performed on Internal Transcribed Spacer 1 (ITS1), selected for the presence in GenBank of numerous sequences belonging to species of the genus *Onopordum*. Other genes (*maturase K* and *trnL-trnF* intergenic spacer region) were not considered given their lower variability. Thirty-four specimens were analyzed: for *O. tauricum*, seven from Sologno, four from Colfiorito, four from Visso, seven from Lecce, four from Rotello, and four from Peschici; for *O. platylepis*, four from Kaiurouan (Tunisia). DNA was PCR-amplified using Platinum Taq (Thermo Fisher Scientific, Carlsbrand, CA, USA); PCR amplification was performed using primers designed by White et al. [121] and Downie and Katz-Downie [122]. PCR products were sequenced using Sanger sequencing technology. The isolated sequences were checked using BLAST and deposited in GenBank (accession numbers: OR941518: OR941551). An additional 18 sequences belonging to the *Onopordum* genus were downloaded from NCBI (see Supplementary Table S4), and the phylogenetic analysis was carried out on a total of 52 sequences using MrBayes-3.2 [123]. ModelTest v. 3.7 (Akaike information criteria, AIC) was employed to determine the best-fit model of DNA substitution: TrN+G. The analysis was performed using all parameter values provided by ModelTest (gamma distribution shape parameter = 0.0150; substitution model: rate matrix A–C 1.0000, A–G 3.0999, A–T 1.0000, C–G 1.0000, C–T 8.4035, G–T 1.0000, base frequencies A 0.2124, G 0.2943, C 0.2902, T 0.2031). The Markov chain Monte Carlo was run for 2,000,000 generations, sampling every 100 generations (burn-in = 25%). Stationarity was defined as when the standard deviation of split frequencies reached 0.008305. The sequence of *Olgaea nidulans* was used as an outgroup.

### 4.6. Morphometric Analysis

For the morphometric analyses, the measurements were taken on the same individuals as those used for the genetic analyses. In total, 33 traits were considered (Table 4). The traits regarding the whole plant were measured on the field, while data regarding leaves, flower heads, stem wing, and achenes were recorded in the laboratory. Indeed, for each individual, three leaves and three flower heads were collected. The leaves were pressed, dried, and scanned using an Epson GT-15000 scanner (Epson America, Inc., Los Alamitos, CA, USA) with a resolution of 600 dpi, capturing both leaf surfaces (abaxial and adaxial) in order to observe the occurrence of protective trichomes and glandular trichomes across the whole leaf. Three flower heads were collected for each plant: the principal one and two flower heads from the lateral branches. The three flower heads were each cut into two hemispheric parts and scanned for subsequent measurements. For each plant, 20 achenes were photographed with a Sony Alpha7III camera mounted with a Sony lens FE24-105 mm F4G OSS. All measurements on scanned and photographed images were taken using ImageJ software 1.53t (ImageJ: https://imagej.net/ImageJ, accessed on 18 September 2023).

A matrix of 120 individuals (20 for each population) × 33 traits was obtained. To compare the morphometric characteristics of the six populations and test their differences, the data matrix (count data were preliminarily square root transformed) was subjected to standardized PCA and Permutational Multivariate Analysis of Variance PERMANOVA [124]. After evaluating the homogeneity of group dispersion through the betadisper() function, we applied PERMANOVA to Euclidean distances using the adonis() function. Both functions are part of the ‘Vegan’ R package 2.6.2 [125]. To assess pairwise differences, post hoc tests were performed utilizing the R package pairwiseAdonis [126] with Bonferroni correction.

The Classification/Regression Tree [127] was employed to identify the most predictive and discriminating morphometric traits among the six populations. We performed 10-fold cross-validation ten times to calibrate the model and obtain a robust estimate of accuracy, thereby minimizing potential biases. During this process, we tested various values of the complexity parameter (cp), ranging from 0.01 to 0.05 with an increment of 0.005. This range allowed us to assess the effect of tree complexity on its predictive and discriminative capabilities. We report the mean Overall Accuracy (OA) index along with their respective standard deviations and a cross-validated confusion matrix, representing the error distribution among populations across the ten repetitions.

## Figures and Tables

**Table 1:** Genetic differentiation measure (F_ST_) among populations.

Population	Colfiorito	Visso	Sologno	Peschici	Rotello	Lecce
Colfiorito						
Visso	0.081952					
Sologno	0.132106	0.111729				
Peschici	0.554903	0.517516	0.605728			
Rotello	0.547273	0.510449	0.596142	0.060023		
Lecce	0.534588	0.499018	0.582669	0.073635	0.065404	

**Table 2:** Measures of genetic diversity: nucleotide diversity π, expected heterozygosity (He), and observed heterozygosity (Ho).

Population	π	He	Ho
Colfiorito	0.00211	0.00205	0.00193
Visso	0.00248	0.00242	0.00237
Sologno	0.00165	0.00161	0.00157
Peschici	0.00153	0.00149	0.00127
Rotello	0.00158	0.00154	0.00127
Lecce	0.00156	0.00152	0.00147

**Table 3:** Geographic location of the six sampled Italian populations of *O. tauricum*.

Population	Site of Collection	Date of Collection	Longitudine	Latitudine	Elevation (m a.s.l.)	Distance from the Sea (km)
SOL	Sologno (RE)	28 July 2020	32T0611040	4912096	803	44.84
COL	Colfiorito (PG)	21 July 2020	33T0331639	4769280	773	70.45
VIS	Cupi di Visso (MC)	14 July 2020	33T0346318	4762414	976	60.01
ROT	Rotello (CB)	19 June 2020	33T0503253	4623847	211	19.66
PES	Peschici (FG)	18 June 2020	33T0584720	4640487	67	3.87
LEC	Frigole (LE)	25 June 2020	34T0266130	4477221	12	1.85

**Table 4:** Morphological traits.

Type of Parameter	Code	Descriptive Parameters and Values	Unit of Measure
Whole plant	HoP1	height of the main stem	cm
	HoP2	maximum plant height	cm
	NoLS	number of lateral branches	unit
	NoL	number of leaves	unit
	NoH	number of flower heads	unit
	LoW	length of the stem wing (15)	mm
	LoT	length of the spine of the stem wing (15)	mm
Leaves	NoLb	number of lobes per leaf	unit
	LoL	leaf length	mm
	LoLLb	length of the longest lobe per each leaf	mm
	WoLLb	width of the longest lobe per each leaf	mm
	LoSLb	length of the shortest lobe per leaf	mm
	WoSLb	width of the shortest lobe per leaf	mm
	LOTL	length of the spine (6 per leaf)	mm
	PoL	perimeter of the leaf	mm
	AoL	area of the leaf	mm^2^
	CoL	circularity of the leaf	ratio
	AroL	aspect ratio of the leaf	ratio
	RoL	round of the leaf	ratio
	SoL	solidity of the leaf	ratio
Flower heads	DoH	diameter	mm
	DoR	diameter of the receptacle	mm
	ToR	thickness of the receptacle	mm
	LoTB	length of the spine of the bract (5 per head)	mm
	GHBH	glandular hairs on the bracts	Visual rating
	TBH	trichomes on the bracts	Visual rating
Fruits	Area	Area	mm^2^
	Perim	Perimeter	mm
	Height	Height	mm
	Width	Width	mm
	AR	Aspect Ratio	ratio
	Circ	Circularity	ratio
	Mean	Medium gray	8-bits

## Data Availability

All data generated or analyzed during this study are contained within the article.

## Supplementary Materials

The following supporting information can be downloaded at: https://www.mdpi.com/article/10.3390/plants13050654/s1; Figure S1: FineRADstructure analyses performed on two distinct clusters (A and B) demonstrate that each investigated subpopulation represents a genetically separate entity. Figure S2: Phylogenetic tree. Phylogenetic analysis was performed with Bayesian inference on Internal Transcribed Spacer 1 (ITS1) sequence, a nuclear marker. The number beside the nodes indicates posterior probability values (>0.95). A total of 52 sequences, of which 34 were obtained in the present study, were analyzed. In green, the group of Sologno (S), Colfiorito (C), and Visso (V) (sites located at the central-northern part of Italy) is reported. In light blue, the sequences of Lecce (L), Peschici (P), and Rotello (R) (sites located in the south of Italy) are shown. The ITS1 sequences obtained from *Onopordum platylepis*, originating from the Tunisian sampling site of Kairouan (Kn), are showed in red. The colored red box indicates sequences that formed a distinct cluster. Table S1: Matrix of the results of measurement, counting, and calculated ratios of all 37 traits considered × 120 individuals (20 for each population). Table S2: Significant group comparisons using Pairwise post hoc tests with Bonferroni correction. Significant group comparisons were conducted using pairwise.adonis function from the pairwiseAdonis package [126]. The table presents the degrees of freedom (Df), pseudo-F value, R2, and *p*-value adjusted for multiple comparisons. Table S3: Cross-validated Confusion Matrix (repeated 10 times with 10-fold cross-validation) obtained from a Classification Tree. The table presents the cross-validated confusion matrix obtained from a Classification Tree model. The confusion matrix reports the percentage of correct and incorrect classifications for each predicted population of *Onopordum tauricum* by the model. Table S4: Sequences selected for phylogenetic analysis.
