# Supplementary material for: Morphological and Genomic Differences in the Italian Populations of Onopordum tauricum Willd.—A New Source of Vegetable Rennet
Source: Plants (Basel). 2024 Feb 27;13(5):654. doi: 10.3390/plants13050654 (PMC10934427; doi:10.3390/plants13050654)
Supplement: Supplementary file 1 [file plants-13-00654-s001.zip › Figure S1.pdf]

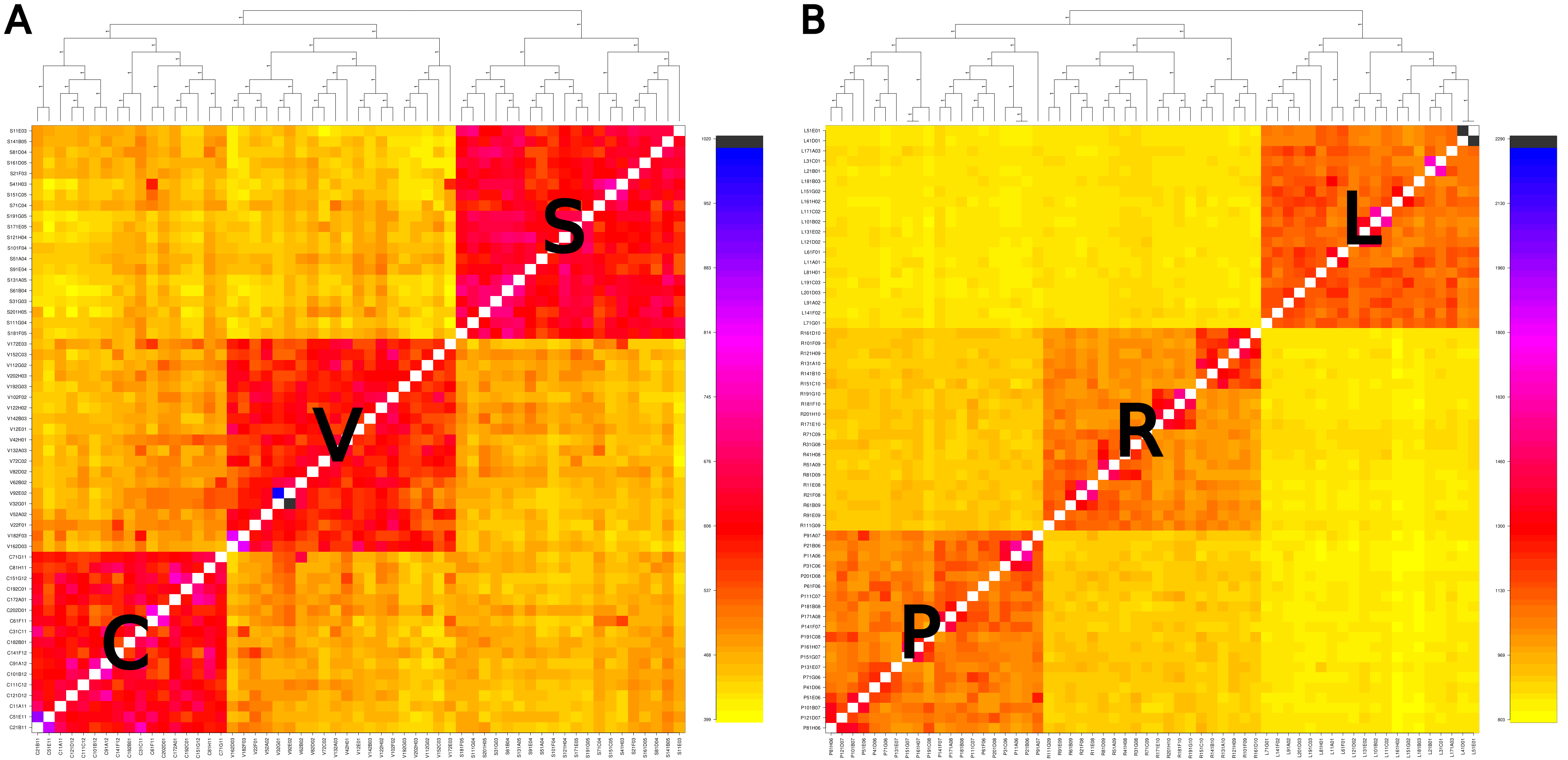
A
S
V
C
B
L
R
P
