# Supplementary material for: Morphological and Genomic Differences in the Italian Populations of Onopordum tauricum Willd.—A New Source of Vegetable Rennet
Source: Plants (Basel). 2024 Feb 27;13(5):654. doi: 10.3390/plants13050654 (PMC10934427; doi:10.3390/plants13050654)
Supplement: Supplementary file 1 [file plants-13-00654-s001.zip › Table S2.docx]

Table S2. Significant group comparisons using Pairwise post hoc tests with Bonferroni correction. Significant group comparisons were conducted using pairwise.adonis function from the pairwiseAdonis package (Martinez Arbizu, 2017). The table presents the degrees of freedom (Df), pseudo-F value, R2, and p-value adjusted for multiple comparisons.

| **Pairs** | **Df** | **Sums of Sqs** | **F.Model** | **R2** | **p.value** | **p.adjusted** |
| --- | --- | --- | --- | --- | --- | --- |
| SOL vs VIS | 1 | 101.58 | 19.44 | 0.34 | 0.0001 | 0.0015 |
| SOL vs COL | 1 | 87.78 | 15.41 | 0.29 | 0.0001 | 0.0015 |
| SOL vs ROT | 1 | 168.84 | 29.12 | 0.43 | 0.0001 | 0.0015 |
| SOL vs PES | 1 | 156.91 | 29.43 | 0.44 | 0.0001 | 0.0015 |
| SOL vs LEC | 1 | 341.27 | 55.13 | 0.59 | 0.0001 | 0.0015 |
| VIS vs COL | 1 | 26.05 | 3.79 | 0.09 | 0.0185 | 0.2775 |
| VIS vs ROT | 1 | 346.9 | 49.77 | 0.57 | 0.0001 | 0.0015 |
| VIS vs PES | 1 | 383.26 | 58.93 | 0.61 | 0.0001 | 0.0015 |
| VIS vs LEC | 1 | 373.71 | 50.76 | 0.57 | 0.0001 | 0.0015 |
| COL vs ROT | 1 | 392.71 | 52.79 | 0.58 | 0.0001 | 0.0015 |
| COL vs PES | 1 | 293.86 | 42.14 | 0.53 | 0.0001 | 0.0015 |
| COL vs LEC | 1 | 423.44 | 54.07 | 0.59 | 0.0001 | 0.0015 |
| ROT vs PES | 1 | 184.31 | 26.05 | 0.41 | 0.0001 | 0.0015 |
| ROT vs LEC | 1 | 119.71 | 15.09 | 0.28 | 0.0001 | 0.0015 |
| PES vs LEC | 1 | 270.72 | 36.25 | 0.49 | 0.0001 | 0.0015 ^1^ |
