# Supplementary material for: Morphological and Genomic Differences in the Italian Populations of Onopordum tauricum Willd.—A New Source of Vegetable Rennet
Source: Plants (Basel). 2024 Feb 27;13(5):654. doi: 10.3390/plants13050654 (PMC10934427; doi:10.3390/plants13050654)
Supplement: Supplementary file 1 [file plants-13-00654-s001.zip › Table S3.docx]

**Table S3.** Cross-validated Confusion Matrix (Repeated 10 times with 10-fold Cross-validation) obtained from a Classification Tree. The table presents the cross-validated confusion matrix obtained from a Classification Tree model. The confusion matrix reports the percentage of correct and incorrect classifications for each predicted population of Onopordum tauricum by the model.

|  | **Reference** | | | | | | |
| --- | --- | --- | --- | --- | --- | --- | --- |
|  |  | LEC | PES | ROT | COL | SOL | VIS |
| **Prediction** | LEC | 13.67 | 1.42 | 1.83 | 0.00 | 0.00 | 0.00 |
|  | PES | 1.75 | 13.92 | 1.92 | 0.00 | 0.00 | 0.00 |
|  | ROT | 1.25 | 1.33 | 12.92 | 0.00 | 0.00 | 0.00 |
|  | COL | 0.00 | 0.00 | 0.00 | 12.17 | 1.58 | 3.33 |
|  | SOL | 0.00 | 0.00 | 0.00 | 1.92 | 14.42 | 2.00 |
|  | VIS | 0.00 | 0.00 | 0.00 | 2.58 | 0.67 | 11.33 |
|  | **OA** | **78.41% (±11.73)** | | | | |  |
