# Supplementary material for: Morphological and Genomic Differences in the Italian Populations of Onopordum tauricum Willd.—A New Source of Vegetable Rennet
Source: Plants (Basel). 2024 Feb 27;13(5):654. doi: 10.3390/plants13050654 (PMC10934427; doi:10.3390/plants13050654)
Supplement: Supplementary file 1 [file plants-13-00654-s001.zip › Table S4.docx]

Table S4. Sequences selected for phylogenetic analysis.

| **Genus** | **Species** | **Accession numbers** |
| --- | --- | --- |
| *Olgaea* | *nitidulans* | FJ007879 |
| *Onopordum* | *acanthium* | MN918976 |
| *Onopordum* | *acaulon* | AF443676 |
| *Onopordum* | *anatolicum* | FJ007884 |
| *Onopordum* | *arenarium* | KX588157 |
| *Onopordum* | *carduchorum* | FJ007885 |
| *Onopordum* | *caricum* | FJ007886 |
| *Onopordum* | *dissectum* | KX588155 |
| *Onopordum* | *hinojense* | KX588153 |
| *Onopordum* | *horridum* | KY418162 |
| *Onopordum* | *illyricum* | MW424424 |
| *Onopordum* | *macracanthum* | KX588151 |
| *Onopordum* | *magrebiense* | KX588150 |
| *Onopordum* | *murbeckii* | KX588149 |
| *Onopordum* | *nervosum* | AY826308 |
| *Onopordum* | *nogalesii* | FJ007888 |
| *Onopordum* | *platylepis* | this study, deposited in Genbank (OR941540:OR941543) |
| *Onopordum* | *seravschanicum* | FJ007890 |
| *Onopordum* | *tauricum* | MW424430 this study, deposited in Genbank (OR941518:OR941539, OR941544:OR941551) |
